# Finite element analysis of different fixation methods of screws on absorbable plate for rib fractures

**DOI:** 10.3389/fbioe.2022.960310

**Published:** 2022-07-22

**Authors:** Hang Xue, Zhenhe Zhang, Mengfei Liu, Ze Lin, Yori Endo, Guodong Liu, Bobin Mi, Wu Zhou, Guohui Liu

**Affiliations:** ^1^ Department of Orthopedics, Union Hospital, Tongji Medical College, Huazhong University of Science and Technology, Wuhan, China; ^2^ Division of Plastic Surgery, Brigham and Women’s Hospital, Harvard Medical School, Boston, MA, United States; ^3^ Medical Center of Trauma and War Injuries, Daping Hospital, Army Medical University, Chongqing, China

**Keywords:** finite element, biomechanical analysis, absorbable plate, rib fixation, fracture healing

## Abstract

Multiple rib fractures caused by trauma are common injuries and the internal fixation methods of these injuries have been paid more and more attention by surgeons. Absorbable plates and screws are the effective way to treat rib fractures, but there are no reports on which type of screw fixation method is most effective. In this study, finite element analysis was used to study the effects of five different types of screw fixation methods on anterior rib, lateral rib and posterior rib. The finite element model of the ribs was reconstructed from CT images, and the internal pressure (40 kPa) and intercostal force (30 N) on the surfaces of the ribs were simulated accordingly. An intercostal force of 30 N was applied to the upper and lower surfaces of the ribs to simulate the effect of intercostal muscle force. The pressure of 40 kPa was applied to the inner surface of the ribs, and the normal direction was applied to the inner surface of the ribs. The positive direction was considered inspiratory pressure, and the negative direction was considered expiratory pressure. The results indicate the optimal type of screw fixation on the absorbable plate for rib fractures, and provide a basis and reference for clinical application.

## Introduction

Rib fracture is a relatively common injury, and multiple rib fractures can disrupt normal life of the affected patients (Baiu and Spain, 2019; Coary et al., 2020). In addition, as the global population continues to age, the incidence of rib fractures has increased significantly. At present, a variety of techniques have been used for the surgical fixation of rib fractures with varying recovery effects (Leinicke et al., 2013). The development of absorbable bone plates has received great attention for the treatment of rib fractures and maintenance of normal respiratory functions in the affected patients (Hur et al., 2016; Oyamatsu et al., 2016). The main rib fixation methods include anterior plate, intramedullary fixation, U-shaped plate and absorbable bone plate (Pieracci et al., 2017; Zhang et al., 2019). Nevertheless, the surgical fixation of rib fractures still needs further improvement. Some remain reluctant to surgically fix fractured ribs because of the poor results of internal fixation, the need for secondary removal, and the lack of strong evidence for this approach.

Biodegradable and absorbable bone plates have been used in a wide range of clinical applications, including cranial and maxillofacial fractures, metacarpal and phalanx fractures, ribs and ankle fractures (Chalidis et al., 2020). About 30% of fracture patients treated with traditional metal fixation experience serious complications, including nonunion, exposed plate, infection, and tendon rupture (Marasco et al., 2010). While metal implants help patients with early mobility, less strong implants may facilitate formation of more callus at the fracture site and improve the healing rates. Stress shielding is also a well-known complication of metal plate fixation, which can be mitigated by the use of a less rigid absorbable plate (Denard et al., 2018; Liverani et al., 2021). Similarly, the use of absorbable plates can reduce the risk of infection associated with traditional metal plate fixation. Several studies evaluating the use of absorbable plates and screws for surgical fixation of rib fractures in flail chest patients have shown that the technique is safe and effective (Nolasco-de la Rosa et al., 2015; Waseda et al., 2019). The slow absorption of absorbable plates gradually transfers pressure to the bone, preventing osteoporosis at the fracture site and promoting fracture healing.

Although there are more and more studies on rib internal fixation, to our knowledge, no previous studies have been published on the effect of rib absorbable screw position on fixation. Previous rib models have incorporated some respiratory muscle mechanics, intercostal muscle movement, and fixation failures, but the optimal fixation type of rib fractures with screws on absorbable plates is yet to be examined. To date, there has been no study that simulates the effect of different screw fixation methods for rib fracture fixation using absorbable plates. Therefore, the purpose of this study was to establish a finite element model of the rib fracture and to analyze the optimal fixation position of the screws on the absorbable plate. These results will enable us to improve the internal fixation of rib fractures and provide a valuable reference for clinical work.

## Materials and method

### Clinical data and finite element modeling

**FIGURE 1:**
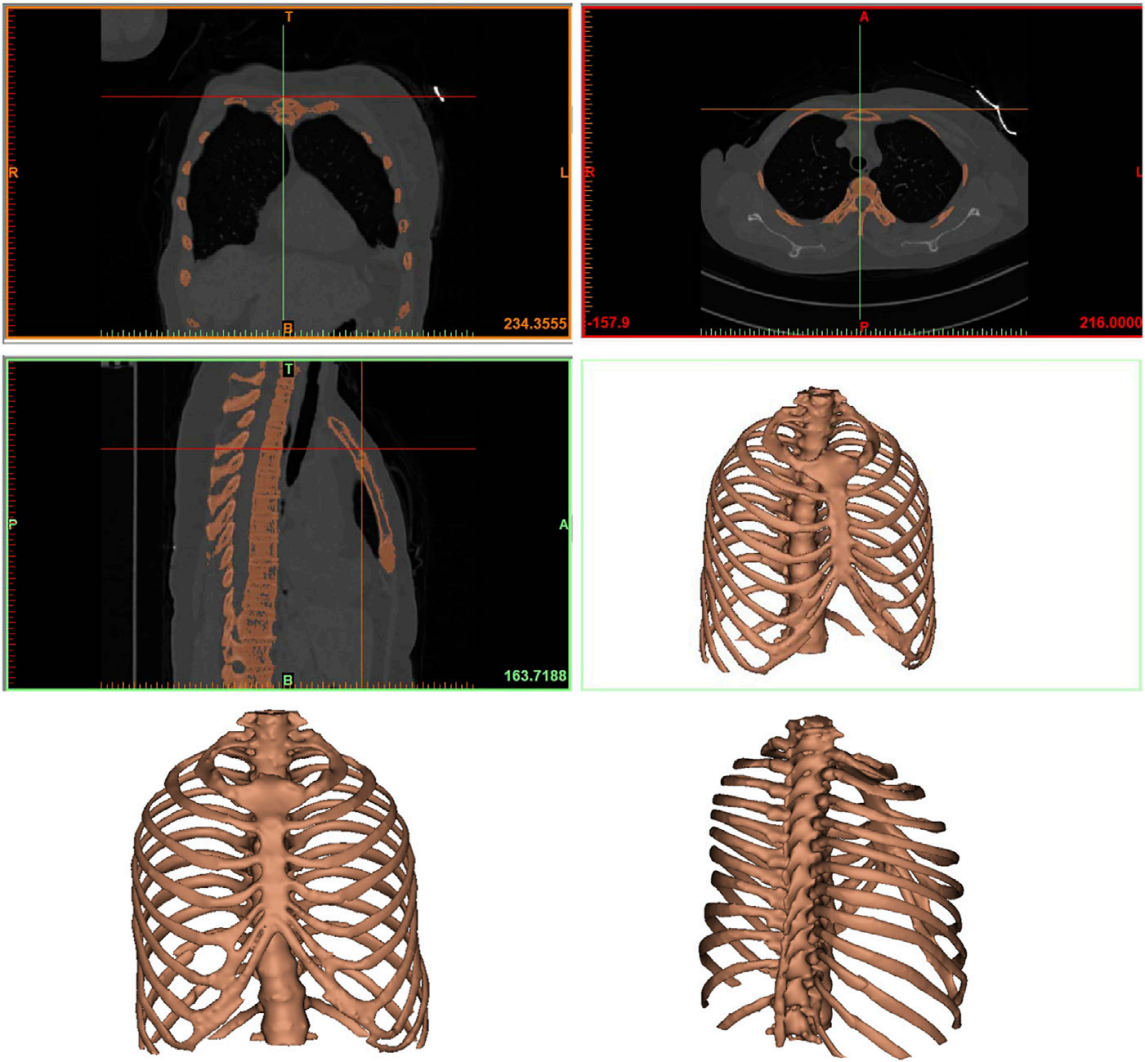
The finite element model of the rib. Anteroposterior and lateral thoracic models were reconstructed from CT data.

A patient with a rib fracture sustained in a car accident underwent a CT examination and modeling after admission. In order to better characterize the injured site of the patient, Mimics software was used to reconstruct the thoracic rib model according to the CT data (Figure 1). A common fracture of the sixth rib was taken as an example to analyze the force of the anterior, lateral and posterior ribs fixed by absorbable plates and screws. Therefore, this part of the study involves construction of a computer finite element model that takes into account the geometry of ribs and plates, material properties and boundary conditions, as well as the respiratory muscle forces.

Firstly, the CT scan image was imported by selecting New Project Wizard on Mimics 20.0 software, and a model was initially generated. The pixels that affect the analysis and the bones that were not used in this analysis were then removed. Calculate 3D tool was used to build a 3D model for each layer, and a rib model was obtained and saved in STL format. Using the Geomagic Studio 2015 software, STL file saved in the previous step was opened and a ×4 mesh subdivision of the model polygon was performed to ensure that no serious deformation would occur during the model processing. The excess deformation features and sharp spikes were removed to obtain an accurate rib model. The precise surface module was used to detect the contour of the model, and the deformed or unreasonable contour was edited. The contour was added appropriately to generate regular surface pieces for the subsequent model processing. After the successful generation of surface slices, the surface slices were fitted, and the fitted surface model was derived into a general Step geometric model format. The Step geometric model file of the rib saved from Geomagic Studio 2015 software was opened with SolidWorks2017 software, and the geometric model was identified and surface was diagnosed according to the software, then the problematic surface was repaired, and the model was saved. The anterior, middle, and posterior fracture models of the ribs were established using a split command, while the ligament force area was cut to facilitate the subsequent loading.

### Experimental conditions and biomechanical analysis

**FIGURE 2:**
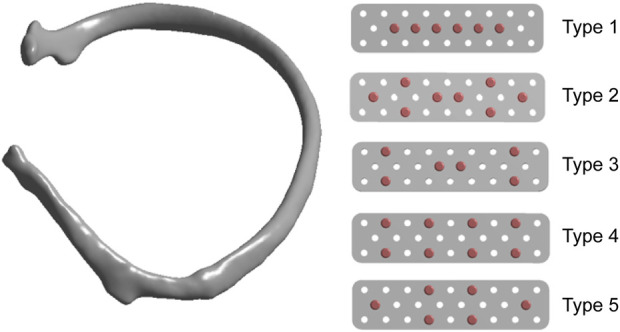
Five different screw fixation methods for the rib fractures with absorbable bone plates.

We set up five types of screw fixation methods on absorbable plates to analyze the stress (Figure 2). Referring to previous literature, in order to facilitate the simulation of real stress state on the ribs, the pressure of respiratory movement was set at 40 kPa, and the force of intercostal muscle was set at 30 N (Marasco et al., 2010; Kang et al., 2018). Establishment of the absorbable plate model: The bone plate and screw were established according to the model parameters (Table 1), and the plate and screw were assembled to the fracture area using the assembly command. After the assembly, the analysis model was saved and imported into ANSYS for finite element analysis. The geometric model was imported into ANSYS19.2, and isotropic material attribute parameters were established respectively, and related materials were assigned to each model. In order to ensure that accuracy of the calculation met the requirements of the analysis, the grid type and size were controlled, and the mesh type was set as the second-order tetrahedral mesh. After the mesh was set, the model was loaded and submitted for calculation according to the pressure and muscle force generated by human respiration.

**TABLE 1:** Mechanical properties of physiological structures and internal fixation implants used to simulate finite element models.

Component/Materials	Elastic modulus (MPa)	Poisson ration
Cortical bone	9000	0.29
Cancellous bone	450	0.29
Cartilage	8	0.40
Absorbable plate/screw	5500	0.30

## Results

**FIGURE 3:**
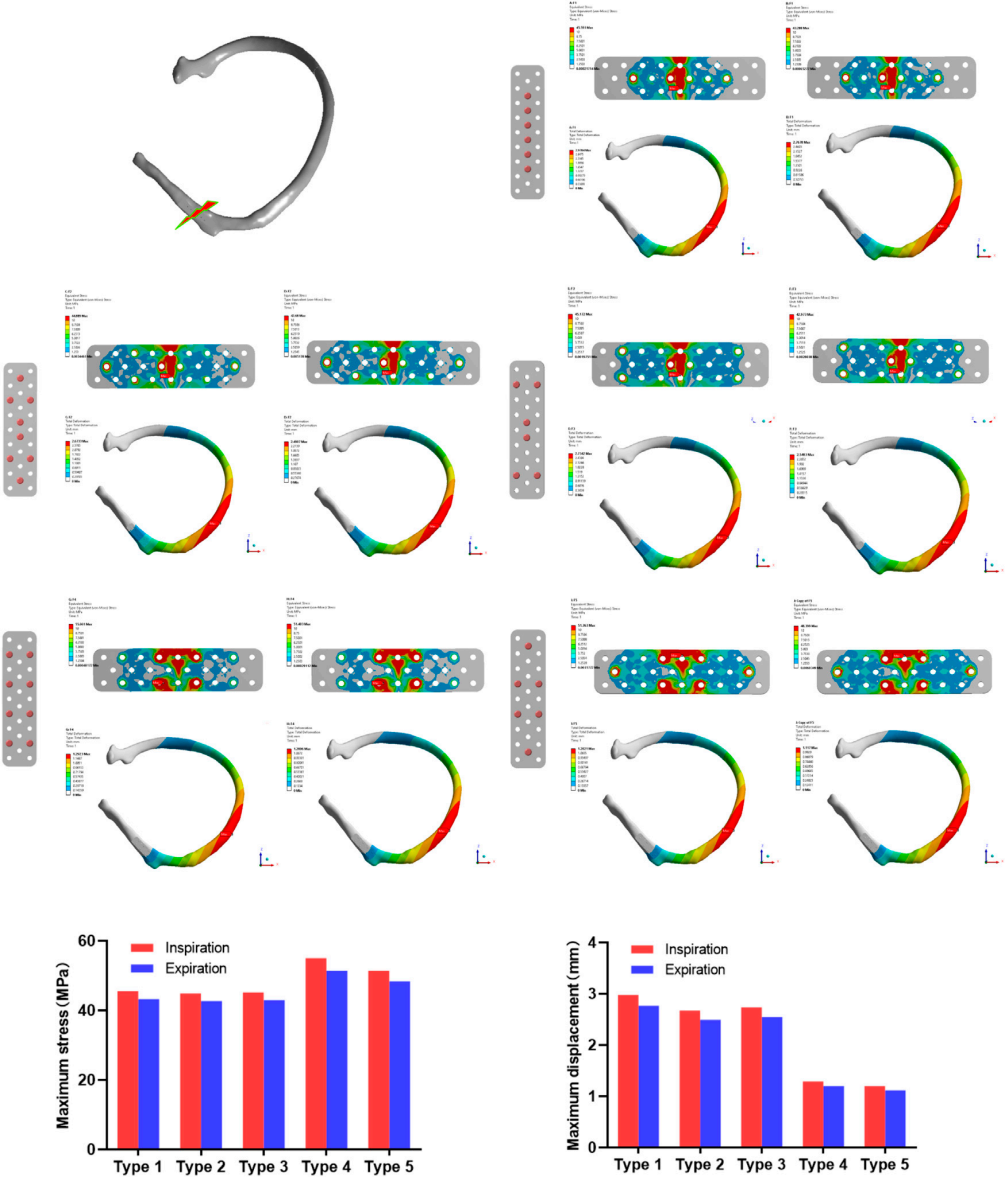
The maximum stress and displacement during inhalation and exhalation with anterior rib fracture in 5 different screw modes after plate fixation.

In this study, finite element analysis was used to simulate the screw distribution during the fixation of rib fracture with absorbable plate, and the optimal screw fixation form was determined to provide a reference for clinical application. In the analysis of the anterior rib fracture, as shown in Figure 3, the maximum stress of the absorbable plate on inhalation and exhalation with the first screw fixation was 45.593 MPa–43.288 MPa, and the maximum displacements during inhalation and exhalation were 2.9784 mm–2.7678 mm, respectively. In the second screw fixation method, the maximum stress of the absorbable bone plate on inspiration was 44.889 MPa and the maximum displacement was 2.6733 mm. During exhalation, the maximum stress of the absorbable bone plate was 42.68 MPa and the maximum displacement was 2.4907 mm. In the third screw fixation method, the maximum stress of the absorbable plate was 45.172 MPa and the maximum displacement was 2.7342 mm on inhalation, while the maximum stress was 42.973 MPa and the maximum displacement was 2.5483 mm on exhalation. In the fourth screw fixation method, the maximum stress of the absorbable bone plate was 55.001 MPa on inhalation and 51.403 MPa on exhalation, and the corresponding maximum displacement of the absorbable plate on inhalation is 1.2923 mm and the maximum displacement on exhalation is 1.2006 mm. In the fifth screw fixation method, the maximum stress and maximum displacement of the absorbable plate on inhalation were 51.363 MPa and 1.2021mm, and the maximum stress and maximum displacement on exhalation were 48.399 MPa and 1.117 mm.

**FIGURE 4:**
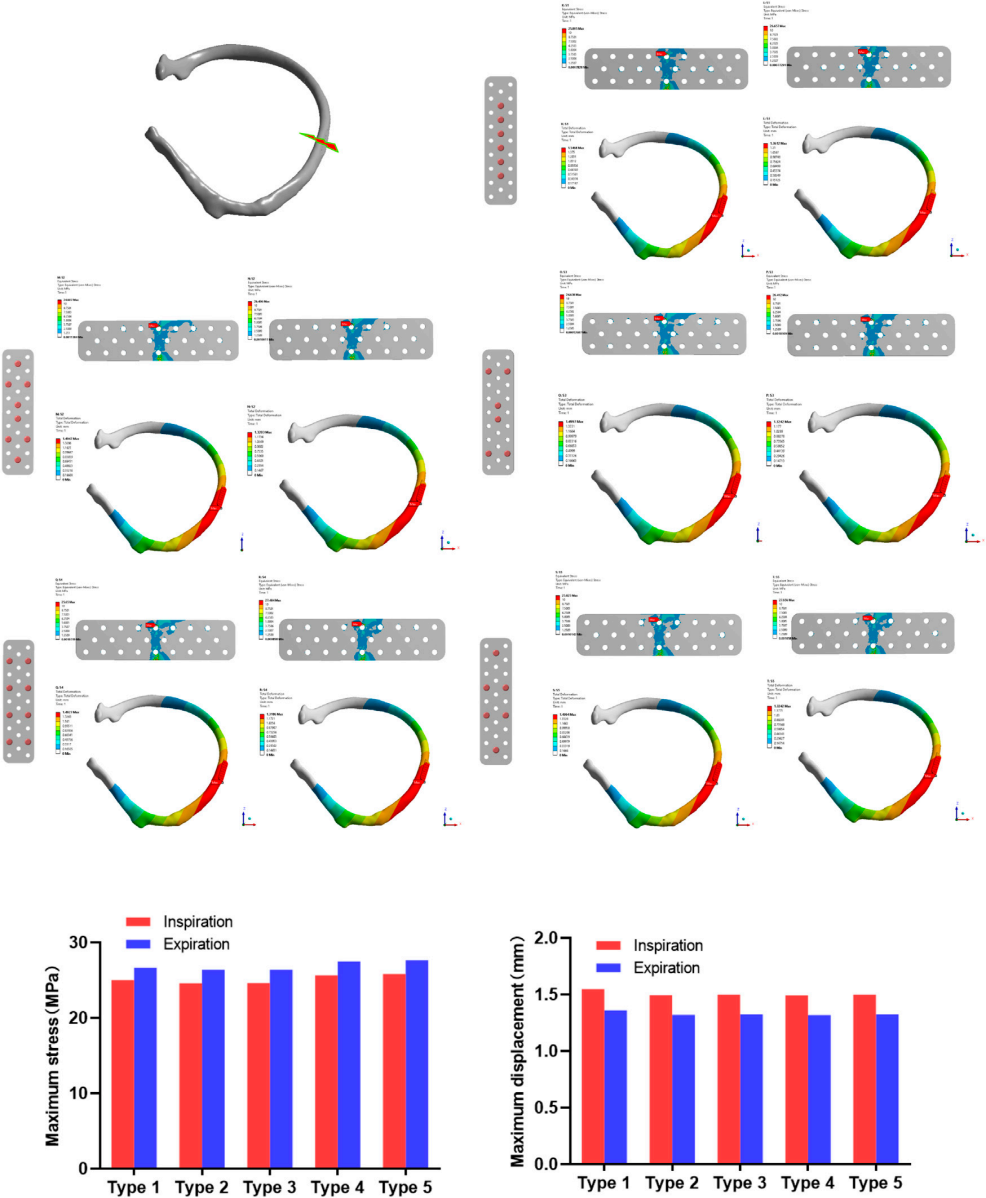
The maximum stress and displacement during inhalation and exhalation after the lateral rib fracture with different screw fixation methods.

In the analysis of lateral rib fracture (Figure 4), the maximum inspiratory stress corresponding to 1-5 screw fixation methods was 25.005, 24.607, 24.638, 25.650, 25.825 MPa respectively. The maximum stress corresponding to expiratory stress was 26.657, 26.406, 26.412, 27.484, 27.656 MPa, respectively. In the analysis of displacement, the maximum displacements corresponding to the 1 to 5 screw fixation modes were 1.5468, 1.4947, 1.4997, 1.4927 and 1.4994 mm during inspiratory, and 1.3612, 1.3203, 1.3242, 1.3186 and 1.3242 mm, respectively during expiratory.

**FIGURE 5:**
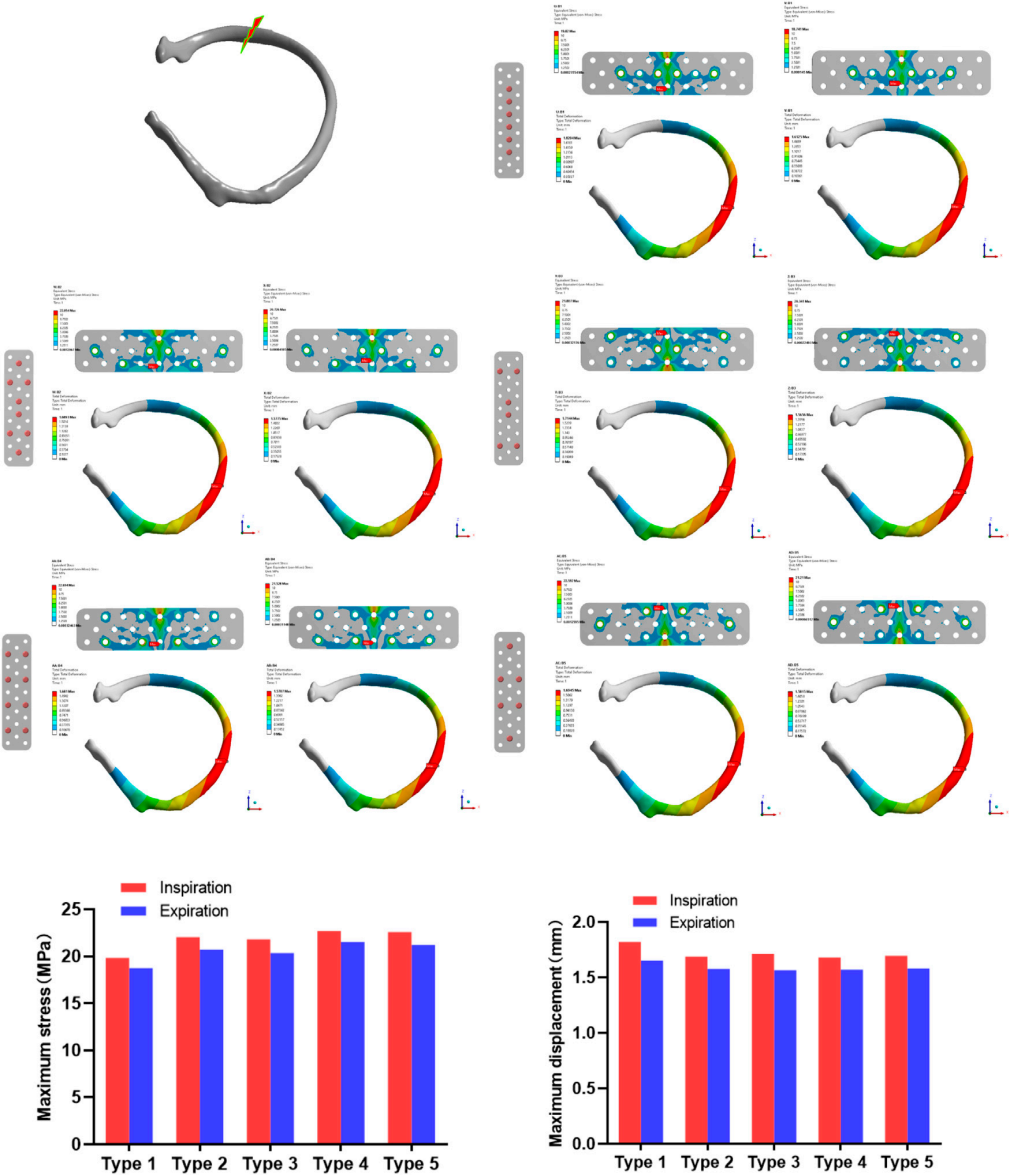
The maximum stress and displacement during inhalation and exhalation of the posterior rib fracture after the fixation with the absorbable plate and screws.

In the stress analysis of the rear rib fracture (Figure 5), the maximum stress of the absorbable bone plate upon inspiration in the 1-5 screw fixation modes was 19.82, 22.054, 21.807, 22.694, and 22.592 MPa. The maximum stress corresponding to exhalation is 18.741, 20.726, 20.341, 21.528, and 21.21 MPa respectively. In the analysis of the displacement of the rear rib fracture, the maximum displacements corresponding to the 1-5 screw fixation modes were 1.8204, 1.6893, 1.7144, 1.681 and 1.6945 mm during inspiration, and 1.6525, 1.5775, 1.5656, 1.5707 and 1.5815 mm respectively during expiration.

## Discussion

The incidence of rib fractures is high, and patients with stable fractures can be treated conservatively with external fixation. In recent years, the treatment of multiple rib fractures has gradually shifted from conservative treatment to surgical internal fixation (Marasco et al., 2012). Early surgery can effectively restore the integrity of the thoracic structure, reduce respiratory energy consumption, accelerate the recovery of patients, and reduce complications (Shen et al., 2008). At the same time, the medical instruments for the fixation of rib fracture are constantly updated, from the early Kirschner wire, intramedullary nail, memory alloy bone graft to claw rib plate and absorbable bone plate, so that the operation is less complicated and the fixation effect is more optimized. The absorbable bone plate is expected to be an ideal rib fixation material because it does not require secondary removal and can eliminate stress masking (Väänänen et al., 2008; Al-Tamimi et al., 2020). Currently, although there are commercial absorbable bone plates for metacarpal, phalangeal, rib and ankle fractures, there is no unified standard for the fixation of screws on bone plates.

In this study, the rib fractures were divided into anterior, lateral and posterior ribs and absorbable plates were fixed according to the five different screw fixation methods described above. In the lateral rib fracture, the screw fixation methods of 1, 4, 5 showed greater stress on the fixed plate, while the 2 and 3 types of screw fixation showed less stress. Except for the displacement of the first fixation method being slightly larger, the displacement gaps of the other four types of screw fixation were not obvious. In the posterior rib fracture, the first type of screw fixation showed less stress, and the other four types of screw fixation showed no significant difference. In the analysis of displacement, the displacement of the first fixed model was more obvious than the other four modes, which was similar to the results of stress analysis.

Compared with the fracture of the lateral rib and the posterior rib, the anterior rib has a weaker structure because of the presence of cartilage, and the bone plate bears most of the force when stressed, so the stress of the anterior rib is relatively large (Holcombe et al., 2020). The analysis results showed that the displacement of the side ribs was relatively small, which may be because the ribs are curved and the structure is relatively stable in the middle of the arc when subjected to internal and external stresses, so the displacement of the lateral ribs fixed with absorbable plates is relatively small.

In summary, the conclusion is that the bone plate with anterior rib fracture has the largest stress, while the bone plates with 4 and 5 type screw fixation methods have the largest stress and the best fixation effect. The 1, 2 and 3 types of screw fixation have less stress, but the fixation effect is not adequate. Based on this, it can be inferred that the fixation effect of the fourth and fifth types of screw is better. Compared with the fourth type, the fifth type uses the least number of screws, so the optimal screw fixation form overall is the fifth type.

## Conclusion

According to the method of finite element analysis evaluating different types of screw fixations of absorbable bone plate, anterior rib fracture fixation showed relatively higher stress values than lateral and posterior rib fractures, especially in the 4th and 5th types of screw fixation methods. Interestingly, 4 and 5 types of screw fixation methods displayed the least displacement for the largest stress value regardless of the type of rib fracture, and the best fixation effect when the stress of the absorbable bone plate was simulated. The fifth fixation method uses fewer screws and therefore can be considered the best fixation method among the five types examined.

## Data Availability

The original contributions presented in the study are included in the article/supplementary material, further inquiries can be directed to the corresponding authors.
